# Impact Comparison of El Niño and Ageing Crops on Malaysian Oil Palm Yield

**DOI:** 10.3390/plants12030424

**Published:** 2023-01-17

**Authors:** Jen Feng Khor, Lloyd Ling, Zulkifli Yusop, Ren Jie Chin, Sai Hin Lai, Ban Hoe Kwan, Danny Wee Kiat Ng

**Affiliations:** 1 Centre of Disaster Risk Reduction (CDRR), Lee Kong Chian Faculty of Engineering & Science, Universiti Tunku Abdul Rahman, Jalan Sungai Long, Kajang 43000, Malaysia; 2 Centre for Environmental Sustainability and Water Security, Universiti Teknologi Malaysia, Skudai 81310, Malaysia; 3 Department of Civil Engineering, Faculty of Engineering, University of Malaya, Kuala Lumpur 50603, Malaysia

**Keywords:** oil palm, ageing crops, FFB crop yield, El Niño-free study, sustainability

## Abstract

Ageing oil palm crops show a significant correlation with the declining oil palm yield in Malaysia. Not only do aged crops result in lower production, but they are also more costly and difficult to harvest. The Malaysian oil palm yield recovered to the pre-El Niño level after the 1997/98 El Niño event. However, the oil palm yield failed to recover after the recent 2015/16 El Niño. Due to the accumulation of aged oil palm plantations in Malaysia, the financial losses from different magnitudes of El Niño events are increasing. Thirty-four years of monthly oil palm yield trends in Malaysia were compared with the El Niño–free yield dataset to show that the oil palm yield downtrend pattern is the same with or without El Niño events in Malaysia for the most recent 15 years (2005 to 2019). The performance of oil palm yield did not show any significant difference from 2000 to 2019. This study estimates that ageing oil palms would lead to a minimum opportunity loss of USD 431 million by December 2022. Without a proper replanting program, the total combined loss attributable to the ageing crops from 2009 to 2022 is estimated to be USD 3.94 billion, which is more profound than losses due to El Niño events within the same period. This study also concluded that a continuous 7-year replanting scheme of at least 115,000 hectares per year is needed to address the adverse impact of ageing crops on the Malaysian oil palm yield, which accounts for nearly 30% of the global palm oil production.

## 1. Introduction

Vegetable oils have one of the highest trade shares in agricultural commodities worldwide, contributing up to 40% of the total production [1]. Among them, palm oil contributes 73 million tonnes, or 35% of the global production of vegetable oil [2]. The domination of palm oil in the vegetable oil sector is due to its high productivity. Palm oil has five times greater yield per hectare than any other oil crop, making it the most productive oil crop in the global market. From a smallholder agroforestry crop, oil palm has developed into the world’s greatest and most crucial vegetable oil over the last 100 years. It is estimated that palm oil alone is capable of fulfilling the enormous and growing demand for vegetable oils worldwide, reaching 420 million tonnes by 2050 [3,4,5,6].

Being the second largest palm oil producer in the world, Malaysia supplies 27% of the total production [7,8,9]. The palm oil industry is a major component of the Malaysian economy. In 2010, the Malaysian government listed the palm oil industry as one of the potential economic sectors that will help the country attain the status of a high-income country under the Economic Transformation Programme (ETP) [10]. In 2021, export revenue from the palm oil industry contributed to 6.5% of the Gross Domestic Product (GDP) in Malaysia [11,12,13]. Furthermore, the palm oil industry is one of the major contributors to job creation and poverty alleviation in Malaysia [3,10,14,15].

**Figure 1:**
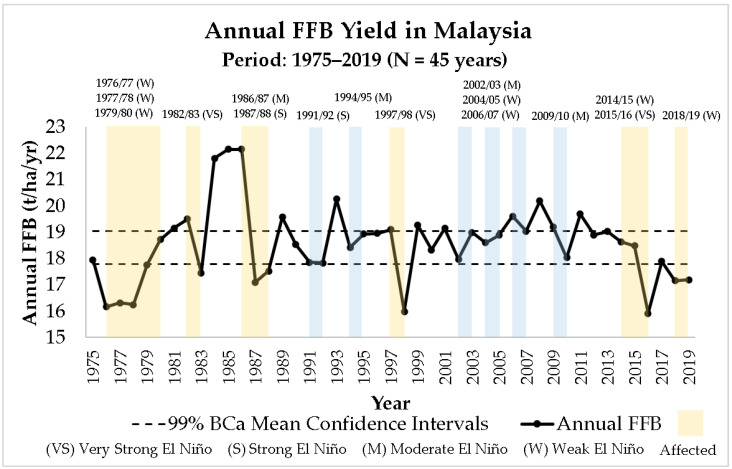
Annual FFB yield in Malaysia from 1975 to 2019 and corresponding El Niño events [7,8,9,24,25].

**Figure 2:**
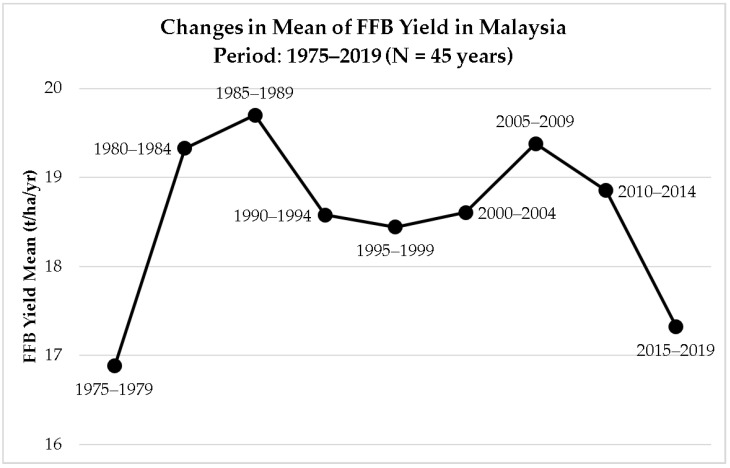
Changes in mean of FFB yield in Malaysia from 1975 to 2019 in moving 5-year intervals [7,8,9].

In the last decade, concerns were raised over the stagnating oil palm yield performance in Malaysia [1,6,16,17,18,19,20,21,22,23]. Based on the historical 45-year records of annual Fresh Fruit Bunches (FFB) yield in Malaysia (Figure 1) [7,8,9], the oil palm yield grew over time until 2008. The unexpected break from the long-term national growth trend in 2009 persisted until the present day, indicating that a new and potentially complicated paradigm was reached [23]. The change in trend is shown clearly in Figure 2, where the FFB yields are computed using the mean of moving 5-year intervals. Over the last 10 years, there was an apparent downward trend in the Malaysian oil palm yield production, approaching the level of the historically lowest point in 1975 to 1979.

The current downward trend in Malaysian oil palm production was forecast by an oil palm predictive model in our past study [26]; however, the reason for the under-yield performance was not explored in the earlier stage of our research. The model predicted monthly oil palm yields and matched closely with the 15 most recent months (July 2021 to September 2022) of reported data in Malaysia.

One of the well-known factors that disrupts agricultural systems, including oil palm, is climate variability. Adverse weather conditions associated with the occurreces of El Niño resulted in the decline of oil palm yields, as El Niño causes extreme hot weather and less rainfall in Malaysia [26,27,28,29,30,31]. Other than the climatic factor, various studies have suspected ageing of oil palm crops as one of the major threats causing the sluggish yield performance in Malaysia in the last decade [1,6,16,17,18,19,20,21,22,23]. However, previous studies lack quantification of the impact of ageing crops on the palm oil industry in Malaysia.

This study aims to identify the underlying factors causing the downslide of oil palm yield in Malaysia. The correlation between aged oil palm crops and the stagnating national FFB yield is established. Quantification analyses are conducted to estimate the losses caused by the ageing oil palm crops. By quantifying the impact of ageing crops, this study hopes to guide stakeholders in the right direction so that the competitive edge of the Malaysian palm oil industry on the global scale can be enhanced.

## 2. Materials and Methods

### 2.1. Statistical Data Collection

Monthly oil palm yield data were collected from the Malaysian Palm Oil Board (MPOB). The MPOB is the premier federal government agency serving the palm oil industry in Malaysia. The MPOB was established on 1 May 2000 as the merger of the functions of the Palm Oil Research Institute of Malaysia (PORIM) and the Palm Oil Registration and Licensing Authority (PORLA) [32]. Yield data from 1975 to 1999 were collected from the PORLA (later became MPOB) annual reports [7], while the data from 2000 to 2020 were collected from the MPOB annual reports [8]. All of the reports are available in both MPOB libraries located at Kajang, Selangor, Malaysia [33], and Kelana Jaya, Selangor, Malaysia [34]. Data from the last 2 years were downloaded from the MPOB website [9]. Table 1 shows the summary of the data collected and used in this study.

### 2.2. Data Processing and Visualization

Statistical analyses were performed to describe the features of the dataset (FFB yield, OER, and CPO price) used in this study, such as distribution, mean, and median. Normality tests were conducted beforehand to determine the central tendency (mean or median) to be used. In this study, the Kolmogorov–Smirnov (KS) and Shapiro–Wilk (SW) tests were used. If the *p*-value is greater than 0.05, then the dataset is considered to be normally distributed, and therefore, mean is used to measure the central tendency of the dataset. On the other hand, if the *p*-value is smaller than 0.05, the dataset assumes non-normal distribution, which uses median as the measure of central tendency [36]. Table 2 shows the normality for the dataset used in this study based on the KS and SW normality tests. The analyses were conducted using IBM SPSS Statistics 26 [37].

#### 2.2.1. Trend Analyses of Annual FFB Yield in Malaysia

To extract the underlying pattern of the oil palm production in Malaysia, trend analyses were conducted. The trend of annual FFB yield from 1975 to 2019 (Figure 1) was plotted chronologically on the graph using Microsoft Excel [38]. The FFB yield data was bootstrapped using Bias Corrected and Accelerated (BCa) procedure (2000 random sampling with replacement). The method neither assumes data normality, nor has limitations to certain data distributions. In this study, the Mersenne Twitter seed number for random sampling generation was set at 2 million [39,40]. All statistical analyses were done using IBM SPSS Statistics 26 [37]. The bootstrapped BCa 99% confidence interval for the mean of 45 years of FFB yield in Malaysia was incorporated in the trend analysis to identify the under-performing years (FFB yield below the lower limit) in the Malaysian palm oil industry.

From 1975 to 2019, the years affected by El Niño events [24,25] were also incorporated in the trend analyses to investigate other factors that affected the oil palm production in Malaysia, other than climatic factor. As discussed in previous works [26], El Niño is a major factor affecting the oil palm yield in Malaysia, as it causes stress to the oil palm trees due to extreme hot weather. There are four categories of El Niño events, which are weak (W), moderate (M), strong (S), and very strong (VS) El Niño [24,25].

To further analyze the trend and performance of the Malaysian palm oil industry, the mean of FFB yield was computed in a 5-year interval moving timeframe, i.e., 1975–1979, 1980–1984, 1985–1989, 1990–1994, 1995–1999, 2000–2004, 2005–2009, 2010–2014, and 2015–2019 (Figure 2). By doing so, the changes in trend of the Malaysian oil palm yield can be shown. Thus, the period in which the trend of FFB yield started to deviate from the overall trend could be pinpointed.

#### 2.2.2. Trend Analyses of El Niño–Free Monthly Dataset in Malaysia

The trend of FFB yield in Malaysia was further analyzed in this study by isolating the climatic threat factor from the national yield dataset. A Malaysian El Niño–free FFB yield dataset from January 1986 to December 2019 (N = 298 months) was created in this study by removing the FFB yields affected by all El Niño events (N = 110 months) during the period as declared in [24,25,26] from the actual recorded FFB yield data in Malaysia [7,8,9]. The trend of monthly FFB yield (median values due to the non-normally distributed dataset) with and without El Niño events were developed in timeframes of 1986–1989, 1990–1994, 1995–1999, 2000–2004, 2005–2009, 2010–2014, and 2015–2019 to compare and contrast the differences of Malaysian FFB yield performance with and without the presence of different El Niño events.

### 2.3. Ageing of Oil Palm Crop Analyses

**Figure 3:**
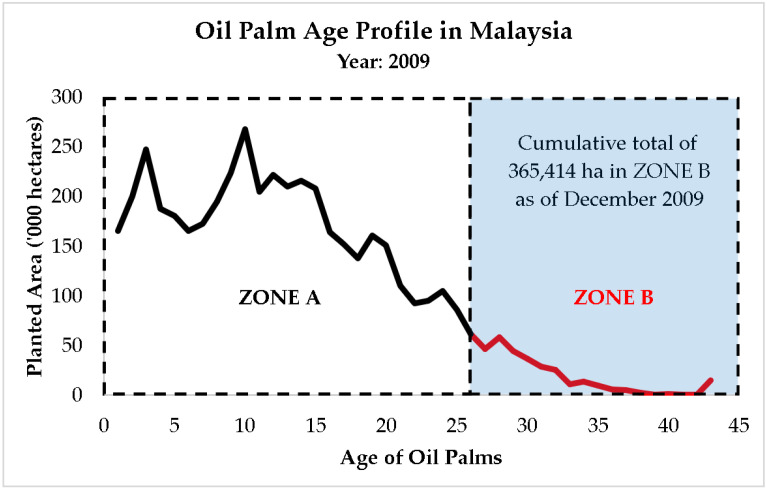
Age profile of oil palm planted area in 2009 [10]. Note: there was no updated age profile reported by government agencies after 2010.

The analyses for the ageing of oil palm crops in Malaysia were conducted using the age profile dataset (Figure 3) published by the Malaysian Prime Minister’s Department in the Performance Management and Delivery Unit (PEMANDU) report in 2010 [10]. In this study, the oil palm crops that exceed 25 years of age were regarded to be aged. According to the age pattern of the oil palm cultivated zone in 2009, as seen in Figure 3, the annual data points of aging oil palm plantation area were traced with the free digitizing software GetData Graph Digitizer v2.26 [41] under this study.

Data points from ZONE A (right to left) represent the upcoming aged oil palm plantation areas in Malaysia that will become more than 25 years old from 2009 onwards (can be projected until 2034). On the other hand, data points from ZONE B (left to right) represent plantation areas that already exceed 25 years old, up to 2009. Those upcoming aged plantation areas from ZONE A will be added to the cumulative areas traced from ZONE B to produce the total accumulated oil palm plantation areas that are at least 26 years old (Figure 3). Those aged plantation areas are classified as under-production areas in Malaysia under this study.

An extensive literature review was performed to obtain a newer age profile. However, updated age profiles after 2010 were not available in official databases. In order for the ageing model to be able to simulate an up-to-date age profile, replanting scenarios were incorporated. This study was able to track the officially published replanting data on the national scale from 2010 to 2013 only. The reported oil palm replanted areas are 116,840 hectares, 102,969 hectares, 113,328 hectares, and 100,852 hectares during the aforementioned four years, respectively [42,43,44,45,46,47]. After this period, the nationwide replanting figures were no longer reported. In order to assess the effectiveness of the replanting scheme, this study incorporated multiple replanting scenarios, including replanting 100,000 hectares/year, 105,000 hectares/year, 110,000 hectares/year, 115,000 hectares/year, 120,000 hectares/year, 125,000 hectares/year, 130,000 hectares/year, 135,000 hectares/year, 140,000 hectares/year, 145,000 hectares/year, and 150,000 hectares/year, to determine the minimum required annual replanting area requirement in Malaysia. The replanting simulation starts from the oldest palm trees available at the modelled year.

#### 2.3.1. Loss Estimation Due to Ageing Oil Palm Crops in Malaysia

The annual FFB loss due to the ageing of oil palm crops can be estimated using Equation (1):

FFB(L) = Σ [FFB_n_−FFB(a)] × A(a), (1)

where FFB(L) is the minimum annual FFB loss due to the ageing of oil palm crops, FFB_n_ is the national average FFB yield, FFB(a) is the FFB yield of the oil palm crops at that age, and A(a) is the aged oil palm plantation area.

The minimum annual opportunity loss due to ageing of oil palm crops can be estimated using Equation (2):

OL(a) = FFB(L) × OER × Price (2)

where OL(a) is the minimum annual opportunity loss due to ageing, OER is the oil extraction rate, and price is the CPO price.

**Figure 4:**
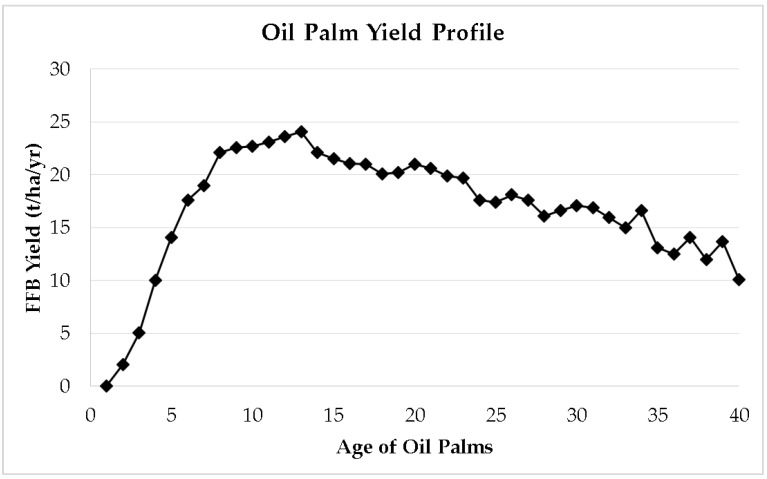
National oil palm yield profile in Malaysia [48].

FFB_n_ from 1986 to 2021 in Malaysia was 18.46 tonnes/hectare/year [7,8,9]. The average OER from 1986 to 2021 in Malaysia was 19.89% [7,8,9]. The average monthly CPO price from 1986 to 2021 in Malaysia was USD 352.38/tonne [35] (1 USD = 4.4496) [13]. FFB(a) is obtained from the national yield profile in Malaysia (Figure 4).

The minimum annual opportunity losses were accumulated at a hypothetical 6% discount rate to show the projected collective impact of ageing oil palm crops. The computation was conducted using the Future Value (FV) function in Microsoft Excel software, where FV is the future value, rate is the discount rate per period, NPER is the total number of payment periods, PMT is the payment made for each period, PV is the present value, and type is the representation of the timing of payment (1 for payment at the beginning of the period, and 0 for payment at the end of the period).

#### 2.3.2. Correlation Test between Aged Area of Oil Palm Crops and Annual FFB Yield in Malaysia

The relationships between the annual area of aged oil palm crops (for all eleven replanting and no replanting scenarios) and annual FFB yield recorded in Malaysia (Figure 1) from 2009 to 2019 were tested using correlation tests. Normality tests were conducted beforehand to determine whether parametric or non-parametric correlation tests should be used. Pearson correlation is a parametric correlation test that measures the linear relationship between two variables that are normally distributed. Spearman’s rho correlation is a non-parametric correlation test that measures the relationship between two variables that are non-normally distributed [49,50,51].

The strength of association between the variables is expressed in a single value between −1 and +1, which is a bivariate correlation analysis. A positive correlation coefficient indicates a positive relationship between the two variables (as values of one variable increase, values of another variable also increase). On the other hand, a negative correlation coefficient expresses a negative relationship (as values of one variable increase, values of another variable decrease). A correlation coefficient with a value of zero indicates that no relationship exists between the variables [26]. All of the tests were conducted using IBM SPSS Statistics 26 [37].

Sensitivity analyses were conducted by repeating the correlation tests for all replanting scenarios in various replanting years, starting from 2010 until 2019, i.e., one replanting year indicated that the replanting scenario was carried out in 2010, two replanting years indicated that the replanting scenario was carried out in 2010 and 2011, and so on. With 11 replanting scenarios in 10 different replanting years, a total of 110 replanting simulations were conducted in this study (refer to Appendix A, Table A1) to search for the minimum required annual replanting scheme.

## 3. Results

### 3.1. Ageing of Oil Palm Crops in Malaysia

#### 3.1.1. Ageing Analysis (No Replanting Scenario)

**Figure 5:**
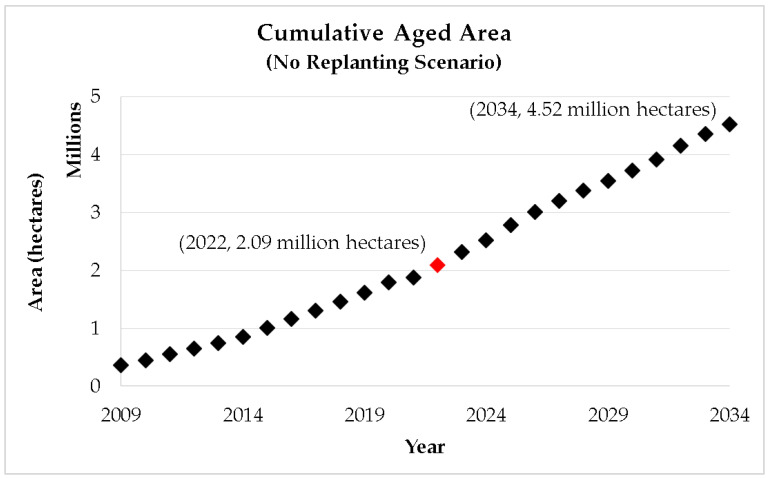
Cumulative hectarage of old oil palm plantation in Malaysia (no replanting scenario).

Based on the Performance Management and Delivery Unit (PEMANDU) report as shown in Figure 3, more than 365,414 hectares of oil palm plantation or 9% of the matured oil palm plantation area exceeded the age of 25 in 2009 [7,10]. These are deemed to be aged crops with lower yield. The total aged area is projected in Figure 5 based on the scenario of zero replanting since 2009. Unless an intervention program is to be implemented, the total aged area will accumulate at the rate of 166,292 hectares/year to reach 4.52 million hectares by 2034, which is nearly 12 times greater than 2009.

By 2022, the accumulated old plantation is estimated to be 2.09 million hectares or about 41% of the 5.14 million hectares of mature oil palm plantation (Table 3). Ageing of oil palm crops has a huge effect on the country’s palm oil output and the sector as a whole. The backlog of old oil palm trees slows down the improvement of crop yield in Malaysia. The adverse effect of the accumulating ageing plantation area deserves a closer study.

**Figure 6:**
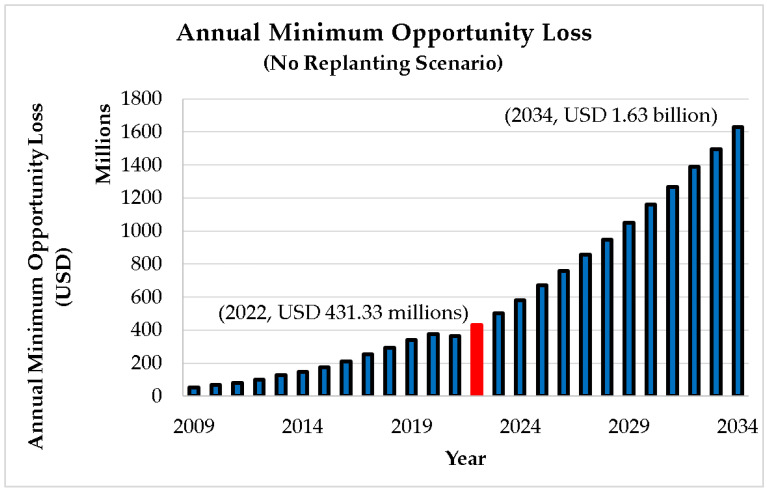
Minimum annual opportunity loss due to ageing of oil palm plantation (no replanting scenario).

Figure 6 shows the minimum annual opportunity loss due to the ageing of oil palm plantation. The rising pattern is followed by a somewhat hyperbolic curve analogous to the trend in the cumulative aged oil palm area. The calculation indicates that the old oil palm will result in a minimum opportunity loss of USD 431 million in 2022 alone, which is roughly 0.12 % of Malaysia’s GDP in 2021 [12,13].

**Figure 7:**
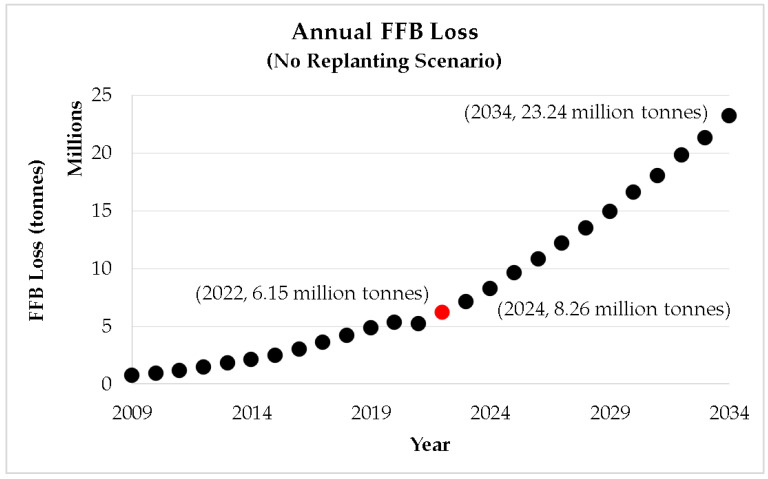
Minimum annual FFB losses due to ageing of oil palm crops (no replanting scenario).

Figure 7 presents the adverse effect of old oil palm trees in Malaysia in terms of the annual FFB production losses. Without any intervention plan, the reduction in FFB production due to ageing crops would reach 8.26 million tonnes in 2024 and surpass the FFB loss caused by the 1997/98 El Niño event (8.18 million tonnes) [26]. Without a proper replanting program, the ageing problem could be described as a continuous hit of El Niño events on the Malaysian oil palm yield every year in the coming years. This issue is too adverse to be neglected. Without strategic management of ageing oil palm crops, Malaysian economic growth would surely suffer.

**Figure 8:**
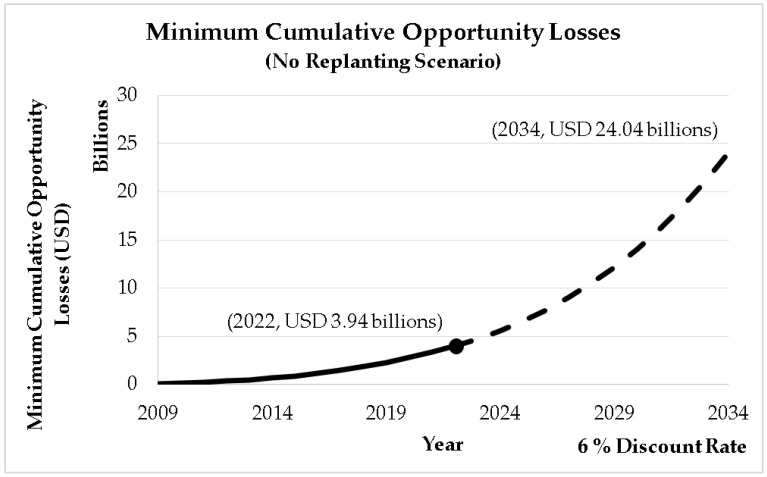
Minimum cumulative opportunity losses due to ageing of oil palm crops (no replanting scenario).

This study then accumulates the annual opportunity losses at a hypothetical 6% discount rate to show the projected collective impact of ageing oil palm crops until 2034, as shown in Figure 8. If no replanting and other interventions are carried out, the accumulated loss due to the ageing crops will increase to a level that would exceed the limits of which the sector is able to cope. From the projected USD 3.94 billion in 2022 (1.1% of Malaysia’s 2021 GDP), the accumulated loss due to the ageing oil palm crops will rise 6.1-fold in the span of 12 years to USD 24.04 billion, which is approximately 6.5% of Malaysia’s GDP in 2021 [12,13].

#### 3.1.2. Comparison of Impact of El Niño and Ageing Oil Palm Crops with Incorporation of Multiple Replanting Scenarios

**Figure 9:**
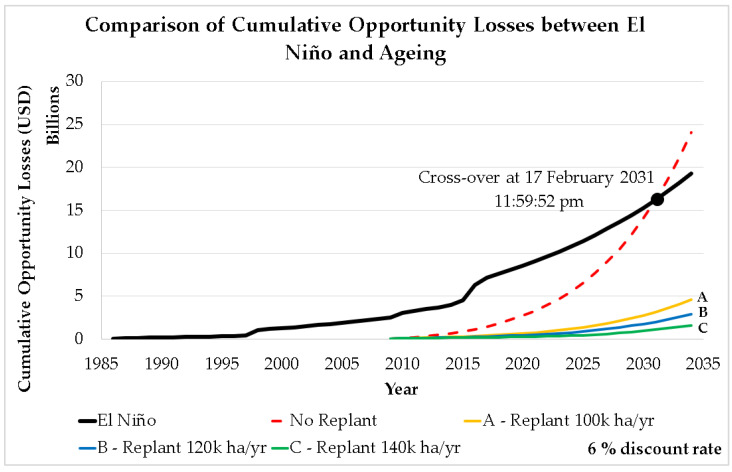
Comparison of cumulative opportunity losses between impact of El Niño and ageing with multiple replanting scenarios [26].

Figure 9 shows the comparison between the minimum cumulative opportunity losses due to El Niño events by previous study [26] and ageing of oil palm crops using a 6% discount rate. Initially, the cumulative losses due to El Niño are higher than that of ageing. The model suggests that the total combined losses attributable to the ageing crops will exceed the losses associated with El Niño events around 2031 if no replanting and other interventions are carried out. By that time, the losses caused by ageing of oil palm crops will be too much for the industry to handle.

Fortunately, it can be seen in Figure 9 that the cumulative losses attributable to the ageing of oil palm crops can be cut down significantly if effective and consistent annual replanting programs are implemented nationwide in Malaysia. Based on the projection up until 2022, the cumulative losses caused by ageing oil palm crops could be reduced by 77, 86, and 92% if replanting activities were carried out in 100,000, 120,000, and 140,000 hectares/year, respectively, since 2009. Delays in carrying out an effective annual replanting program could have dire consequences on the Malaysian palm oil industry. The unattended issue of ageing oil palm crops is slowly eroding the national oil palm yield capacity.

**Figure 10:**
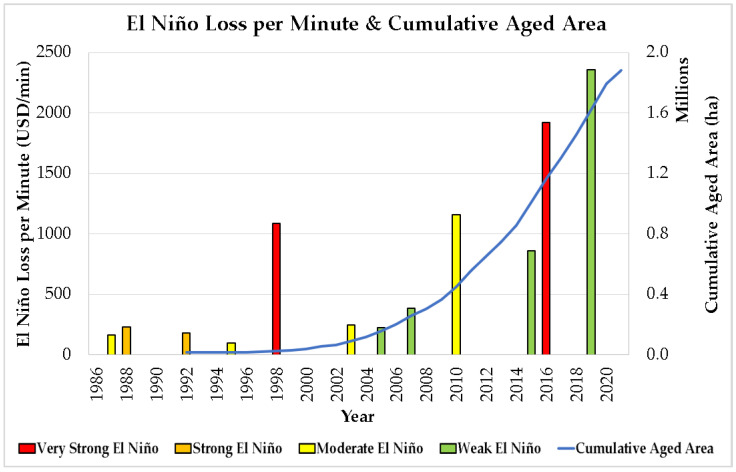
Comparison between El Niño loss per minute and cumulative aged area (no replanting scenario) [26].

Figure 10 shows the comparison between the El Niño losses computed by a previous study [26] in terms of El Niño loss per minute and the cumulative aged area of Malaysian oil palm plantations (Figure 5). In each category of El Niño events that happened from 1986 to 2019, the losses attributable to El Niño increase as the aged area of Malaysian oil palm plantation accumulates.

### 3.2. Correlation between Ageing Crops and Oil Palm Yield in Malaysia

The declining yield in the Malaysian oil palm production from 2009 to 2019 is correlated with the accumulation of aged oil palm plantation areas based on multiple replanting scenario analyses. As shown in Table 4, the ageing of oil palm crops has statistically significant negative correlations with annual FFB yield recorded in Malaysia, with a correlation of −0.715 for no replanting scenario, −0.673 for the replanting scenario of 100,000, −0.711 for the replanting scenario of 105,000, and −0.650 for the replanting scenario of 110,000 hectares/year (significance at 0.05 alpha level).

Simulated replanting scenarios of 115,000 hectares/year and above for the full duration from 2009 to 2019 no longer show significant negative correlation with recorded FFB yield in Malaysia (Table 4). However, based on the sensitivity analyses performed on the correlation tests, at least seven consecutive years of oil palm replanting activities are needed to be carried out for the replanting scenario of 115,000, 120,000, 125,000 and 130,000 hectares/year in order for the national FFB yield trend to cease the negative correlation with ageing of oil palm crops. For replanting scenario of 135,000, 140,000, 145,000 and 150,000 hectares/year, at least six consecutive replanting years are required for the replanting programs to be effective.

**Figure 11:**
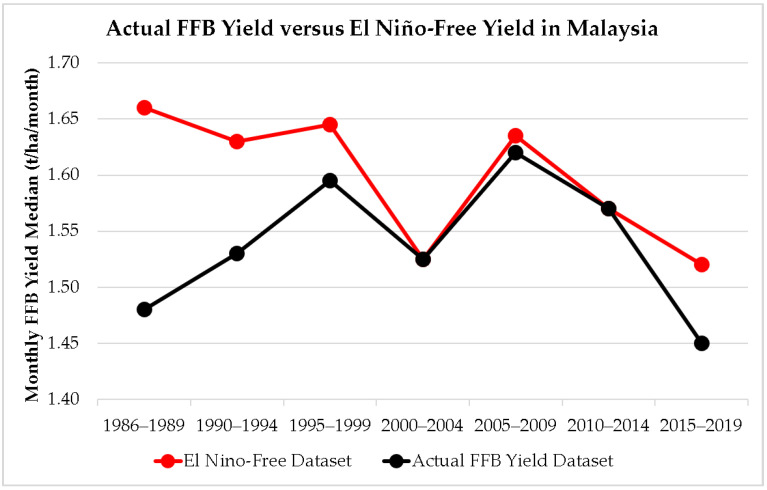
Comparison of monthly recorded FFB yield trend and El Niño–free yield data in Malaysia computed in median values for the aforementioned durations between 1986 and 2019. Actual FFB yield data recorded from January 1986 to December 2019 in Malaysia (N = 408 months), including 110 months of yield data, which are associated with El Niño as declared by [24,25,26], and 298 months of El Niño–free yield dataset.

Figure 11 shows the 34 years of monthly FFB yield trend in Malaysia compared with the El Niño–free yield dataset. The FFB yield downtrend pattern is identical with or without El Niño events in Malaysia for the most recent 15 years (2005 to 2019). When FFB yield associated with El Niño events is excluded from the actual monthly data in Malaysia to perform the El Niño-free dataset analyses, the performance of FFB yield did not show any significant improvement from 2000 to 2019. FFB yield data from January 2020 onward was excluded from these analyses, as Malaysia entered a COVID-19 nationwide lockdown and movement control from March 2020–November 2021. Oil palm harvesting activities were delayed during this period. The presence of yield downtrend patterns even in the El Niño–free dataset in recent 15 years serves as a warning to the Malaysian palm oil industry stakeholders that other hidden threats (ageing of oil palm crops identified as one of them in this study) are present in the industry and are affecting the Malaysian FFB yield performance in a negative way. Malaysian FFB yield was in a downtrend in the last 15 years even without the impact from different magnitudes of El Niño events (Figure 11).

## 4. Discussion

This study identified and quantified the major threat obstructing the oil palm yield growth in Malaysia, which is the ageing of oil palm crops. In light of the only available oil palm age to yield profile, which was published in the ETP annual report 2010, over 365,414 hectares of oil palm plantation were considered to be aged as of the end of 2009 [10]. If the situation is not taken care of, at the end of 2022, the total aged area will increase at the rate of 132,733 hectares/year to reach 2.09 million hectares or 41% of the matured oil palm plantation area in Malaysia. This would cause an estimated opportunity loss of USD 431 million in 2022 or 0.12% of Malaysia’s 2021 GDP [12,13]. Even in a well-managed private corporation, the issue of aged oil palm crops occurs. In 2021, about 35% of the oil palm plantation owned by IOI Group in Malaysia had already passed their prime production age [53].

This study successfully established significant correlations between the ageing of oil palm crops and the declining Malaysian oil palm yield in recent years (Table 4). If the aged areas of oil palm crops in Malaysia are left to accumulate year after year, they will bring down the national oil palm yield further. Over the last decade, ageing crops have been suspected to be the underlying factor in slowing down the oil palm yield growth in Malaysia. In 2012, the United States Department of Agriculture (USDA) reported that more than a quarter of the oil palm plantation in Malaysia has already reached or passed the peak yielding years [23]. Concerns that the decline in national yield statistics would continue were raised and, evidently, were correct. In 2019, Dorab Mistry, an expert in the vegetable oil market, warned that the palm oil production would suffer due to the older age of oil palm plantations in Malaysia [54]. Based on the OECD-FAO Agricultural Outlook published in 2021, it is reported that the growth in the production of palm oil in Malaysia would slow down because of the ageing oil palm crops [55]. However, none of those reports offered a viable solution to curb the FFB yield reduction problem in Malaysia.

The situation becomes worse when extreme weather anomalies, such as El Niño events, hit Malaysia. Aged oil palm trees are definitely not as resilient to the stress caused by El Niño. This effect can be seen in Figure 1, where the Malaysian oil palm yield dropped below the 99% confidence interval just because of a single weak El Niño event in 2018/19. According to the historical 45-year oil palm yield data, the FFB yield recorded in Malaysia never fell below the 99% confidence interval due to weak El Niño, except for the three consecutive weak El Niño events between 1976 and 1980. The Malaysian oil palm yield recovered to the pre-El Niño level after the 1997/98 El Niño event. However, the oil palm yield failed to recover after the recent 2015/16 El Niño, up until today. Following the accumulation of aged oil palm plantations in Malaysia, the financial loss of different magnitudes of El Niño event keeps increasing, as shown in Figure 10.

Over the years, the rapid expansion of oil palm plantations has reportedly caused negative impacts on biodiversity. Deforestation driven by large-scale oil palm cultivation is a significant threat to the local environment and rainforests. Campaigns have been raised against the crop, stating that oil palm cultivation has resulted in the destruction of rainforests and the endangerment of orangutans. A global boycott on palm oil would not be a practical solution. Replacing palm oil with an alternative might worsen the sustainability issue because other oil crops require more land for cultivation. The extent of further negative consequences for tropical biodiversity will depend on the improvement in the production of palm oil. It is essential to improve the sustainability of the palm oil industry, covering all aspects of social, economic and environmetal impact [5,16,56,57,58,59,60,61,62,63,64,65,66,67,68,69].

Improving oil palm yield in existing plantations through replanting old crops is the most responsible way to produce sufficient palm oil for global demand. The age of the palm trees is one of the important components in the production of fruit bunches [70]. When the age of oil palm passes its peak yielding age, the production of fruit bunches decreases [71]. Numerous problems arise when the palm trees become old in a plantation. Oil palm harvesting becomes difficult, resulting in additional labor demand, and it is also harder to assess the bunch ripeness [72]. To avoid the accumulation of lower yielding older palms in plantations, replanting needs to be carried out on a continuous basis using new high-yield seed varieties [18,23,73,74,75,76]. The growth in palm oil production as well as yield improvement depend heavily on the acceleration of replanting activities consistently over years [55].

The correlation tests between aged plantation areas under multiple replanting scenarios and FFB yield in Malaysia (Table 4) show that the ageing of oil palm crops is significantly correlated with the declining yield of Malaysian oil palm. Replanting scenarios below 115,000 hectares/year show significant negative correlations (at alpha = 0.05 level) with the recorded FFB yield in Malaysia, implying that the minimum required nationwide replanting scheme must not be less than 115,000 hectares/year to be effective. Unfortunately, three out of four years (2010 to 2013) of nationwide replanting data published officially noted less than 115,000 hectares/year replanting and, therefore, did not meet the minimum required area to be considered as an effective replanting scheme by this study. This study further conducted El Niño–free analyses to isolate the climatic threat from the FFB yield trend in Malaysia. Shockingly, the El Niño–free FFB yield pattern not only showed an apparent downtrend in the most recent 15 years but also did not demonstrate a significant improvement in the FFB yield data. This infers the presence of a hidden threat due to the ageing of oil palm crops in the declining yield performance in Malaysia apart from climatic factors.

**Figure 12:**
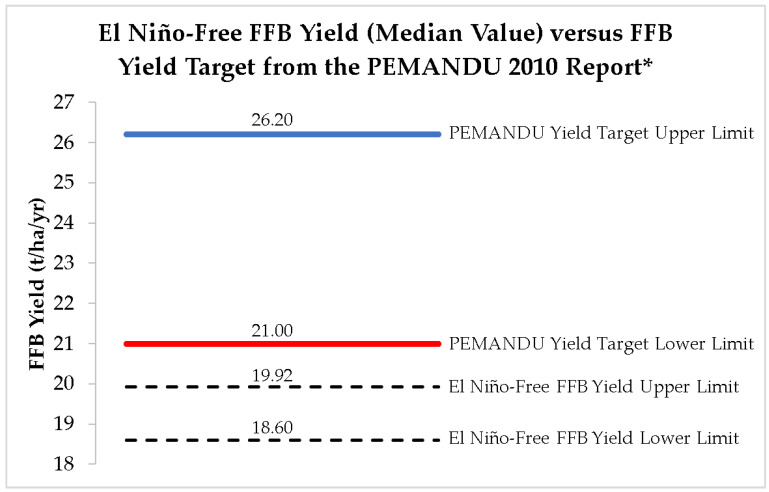
Comparison of PEMANDU FFB yield target and the El Niño–free yield data study (bootstrapped BCa 99% confidence intervals) in Malaysia. * PEMANDU 2010 ETP report can be found in [10].

In the ETP 2010 annual report [10], the PEMANDU agency from the Malaysian Prime Minister’s Office announced the Malaysian oil palm FFB yield improvement targets (in this study, known as PEMANDU yield target lower and upper limits) to be 21.00 and 26.20 t/ha by 2020. The actual recorded oil palm FFB yield was only 17.19 in 2019 and 16.73 t/ha in 2020 [8]. Even in the best nation yield study with the El Niño–free yield dataset from 1986 to 2019, the calculated bootstrap BCa 99% confidence upper limit does not reach the lower limit of the PEMANDU yield target (Figure 12).

Despite advancements in genome research and technology [77,78,79,80], the oil palm yield growth in Malaysia remains sluggish in the last decade, indicating that the replanting programs currently carried out in the country (if any) are not sufficient or effective enough to improve the national FFB yield. As simulated and shown in Figure 9, a consistent nationwide annual replanting scheme could decrease losses caused by ageing crops by a significant amount. Without continous replanting efforts, the cumulative losses attributable to ageing crops will overtake losses associated with El Niño events. The timing and severity of the climate change anomalies could not have been foreseen. However, the issue of ageing crops can be addressed immediately. Hence, there is no excuse for failing to address the issue. Stakeholders in the Malaysian palm oil industry are urged to focus on the right direction and commit to effective and continous replanting efforts.

## 5. Conclusions

Key findings from this study are as follows:

1. Delay in carrying out a replanting program for the existing old oil palm plantation poses dire effects on the Malaysian oil palm industry. If a replanting program is not implemented, the cumulative aged area of the oil palm crops in Malaysia is estimated to reach 2.09 million hectares in 2022, which is 41% of the matured oil palm plantation area. The accumulated loss caused by ageing oil palm crops could increase 6.1-fold, from USD 3.94 billion in 2022 (nearly 6.5% of the Malaysia’s GDP in 2021) to USD 24.04 billion in 2034.
2. This study further conducted El Niño–free dataset analyses to isolate the climatic threat from the FFB yield trend in Malaysia (Figure 11). The El Niño–free FFB yield pattern not only showed an apparent downtrend in the most recent 15 years but also did not demonstrate any significant improvement in the FFB yield data in 2000–2019. This infers the presence of a hidden threat due to the ageing of oil palm crops in the declining yield performance in Malaysia apart from climatic factors. Malaysian FFB yield had a downtrend in the last 15 years (2005 to 2019), even without the impact of different magnitudes of El Niño events.
3. The ageing oil palm crop issue could be controlled by effective replanting schemes, as shown in this study, whereby the losses due to ageing crops could be brought down significantly when replanting plans are consistently implemented over years. Our correlation analysis between aged plantation area and oil palm yield in Malaysia concluded that nationwide replanting programs of less than 115,000 hectares/year are not effective. This study suggested that continous replanting programs of at least 115,000 hectares/year for a minimum of 7 years are needed to mitigate the impact of ageing crops on the low yield of Malaysian oil palm production. Without continous replanting efforts, the cumulative losses attributable to ageing crops will overtake the losses associated with El Niño events. The timing and severity of the climate change anomalies could not have been foreseen. However, the issue of ageing crops can be addressed immediately in Malaysia.
4. The primary aim of the replanting initiative is to replace underperforming oil palms aged over 25 years old. Terrain cultivation for oil palm plantation is at its height in Malaysia, which is why it is also important to use new high-yields seed varieties in future replanting programs. Meanwhile, replanting programs must be carried continuously over years to ensure the sustainability of Malaysian FFB yield in future.
5. This study could be strengthened and enhanced by using an updated oil plam age to yield profile for more accurate FFB yield projections in Malaysia, Indonesia, and Thailand. Additional variables that could influence the palm oil production can be incorporated in future research. This encompasses the trajectory in the commercialization of land conversion in Malaysia, because in recent years, several private oil palm plantation estates have been converted into commercial and residential areas in Malaysia. Other means to improve the sustainable cultivation of oil palm should also be studied further.

## Figures and Tables

**Table 1:** Summary of the Malaysian oil palm statistical data collected and used in this study.

Variable(s)	Unit	Periodicity	Duration	Source(s)
Fresh fruit bunches (FFB) yield	tonnes/hectare (t/ha)	Yearly	1975–2021 (N = 47 years)	[7,8,9]
Oil palm planted area	hectares (ha)	Yearly	1975–2021 (N = 47 years)	[7,8,9]
Oil extraction rate (OER)	percentage (%)	Yearly	1986–2021 (N = 36 years)	[7,8,9]
Crude palm oil (CPO) price	United States Dollar/tonne (USD/t)	Monthly	January 1986–December 2021 (N = 432 months)	[35]

FFB are the raw products harvested from the oil palm trees, while CPO is the oil extracted from the flesh of the fruit [5]. The monthly CPO price, which was originally in Malaysian Ringgit/tonne (MYR/t), was converted to USD (1 USD = 4.4496 MYR) [13].

**Table 2:** Normality tests for the dataset used in this study.

Variable(s)	KS *p*-Value	SW *p*-Value	Normality
FFB yield	0.2000 (>0.05)	0.0880 (>0.05)	Normal
OER	0.0321 (<0.05)	0.0074 (<0.05)	Non-normal
CPO price	0.0000 (<0.05)	0.0000 (<0.05)	Non-normal

**Table 3:** Oil palm planted area (hectare) in Malaysia as of December 2021 [52].

State	Matured (Prime and Old)		Immature (Young)		Total	
	(Hectare)	(%)	(Hectare)	(%)	(Hectare)	(%)
Johor	652,568	93.3	46,648	6.7	699,217	12.2
Kedah	76,071	87.5	10,915	12.5	86,986	1.5
Kelantan	136,943	83.4	27,336	16.6	164,279	2.9
Melaka	50,219	92.8	3913	7.2	54,131	0.9
Negeri Sembilan	172,151	93.2	12,524	6.8	184,674	3.2
Pahang	686,560	90.8	69,346	9.2	755,906	13.2
Perak	335,998	91.1	33,021	8.9	369,018	6.4
Perlis	758	99.8	2	0.2	760	0.0
Pulau Pinang	9522	98.3	161	1.7	9684	0.2
Selangor	100,495	91.2	9754	8.8	110,250	1.9
Terengganu	142,585	82.4	90,357	17.6	172,942	3.0
Peninsular Malaysia	2,363,870	90.6	243,977	9.4	2,607,847	45.5
Sabah	1,331,981	87.4	191,643	12.6	1,523,624	26.6
Sarawak	1,448,329	90.2	157,932	9.8	1,606,261	28.0
Sabah and Sarawak	2,780,310	88.8	349,574	11.2	3,129,884	54.5
Malaysia	5,144,180	89.7	593,551	10.3	5,737,731	100.0

**Table 4:** Correlations between aged area and FFB yield from 2009 to 2019 (refer to Appendix A, Table A1 for further details).

Replanting Scenario	Correlation Coefficient	*p*-Value	Replanting Years Needed to Break the Correlation with Declining Yield
No replanting (Figure 5)	−0.715 ^a^	0.0135 *	-
Replant 100,000 hectares/year	−0.673 ^b^	0.0233 *	0
Replant 105,000 hectares/year	−0.711 ^b^	0.0142 *	0
Replant 110,000 hectares/year	−0.650 ^a^	0.0305 *	0
**Replant 115,000 hectares/year**	−**0.577 ^a^**	**0.0631**	**7**
**Replant 120,000 hectares/year**	−**0.440 ^a^**	**0.1751**	**7**
**Replant 125,000 hectares/year**	−**0.225 ^a^**	**0.5052**	**7**
**Replant 130,000 hectares/year**	**0.022 ^a^**	**0.9494**	**7**
**Replant 135,000 hectares/year**	**0.225 ^a^**	**0.5058**	**6**
**Replant 140,000 hectares/year**	**0.362 ^a^**	**0.2737**	**6**
**Replant 145,000 hectares/year**	**0.418 ^b^**	**0.2006**	**6**
**Replant 150,000 hectares/year**	**0.518 ^b^**	**0.1025**	**6**

* Correlation is significant at α = 0.05. ^a^ Pearson correlation coefficient (for normally distributed variables). ^b^ Spearman’s rho correlation coefficient (for non-normally distributed variables).

## Data Availability

Oil palm data can be found in Palm Oil Registration & Licensing Authority (PORLA) reports “PORLA palm oil statistics” from 1986 to 1999 and Malaysian Palm Oil Board (MPOB) reports “Malaysian oil palm statistics” from 2000 to 2020 in MPOB libraries located in Kelana Jaya and Bangi, Malaysia. Recent oil palm data are available at “https://bepi.mpob.gov.my/index.php/en/ (accessed on 17 November 2022)”. MPOB is the federal government agency established in 2000 to serve the Malaysian palm oil industry. Prior to this, it was known as the PORLA agency. The data presented in this study are available upon request from the corresponding author.

## Appendix A

**Table A1:** Correlations between aged area and FFB yield from 2009 to 2019 for multiple replanting scenarios.

Replanting Scenario	Replanting Year(s) Starting from 2010									
	1	2	3	4	5	6	7	8	9	10
Replant 100,000 hectares/year	−0.714 *	−0.735 *	−0.734 *	−0.736 **	−0.673 *	−0.673 *	−0.673 *	−0.673 *	−0.673 *	−0.673 *
Replant 105,000 hectares/year	−0.714 *	−0.735 **	−0.734 *	−0.765 **	−0.711 *	−0.711 *	−0.711 *	−0.711 *	−0.711 *	−0.711 *
Replant 110,000 hectares/year	−0.714 *	−0.736 **	−0.734 *	−0.791 **	−0.736 **	−0.700 *	−0.700 *	−0.700 *	−0.700 *	−0.650 *
Replant 115,000 hectares/year	−0.713 *	−0.736 **	−0.734 *	−0.791 **	−0.718 *	−0.645 *	−0.564	−0.564	−0.564	−0.577
Replant 120,000 hectares/year	−0.713 *	−0.737 **	−0.734 *	−0.791 **	−0.718 *	−0.627 *	−0.509	−0.455	−0.455	−0.440
Replant 125,000 hectares/year	−0.713 *	−0.738 **	−0.734 *	−0.717 *	−0.718 *	−0.627 *	−0.418	−0.364	−0.299	−0.225
Replant 130,000 hectares/year	−0.713 *	−0.738 **	−0.733 *	−0.714 *	−0.661 *	−0.627 *	−0.355	−0.227	−0.151	0.022
Replant 135,000 hectares/year	−0.713 *	−0.739 **	−0.733 *	−0.710 *	−0.651 *	−0.546	−0.273	−0.272	0.005	0.225
Replant 140,000 hectares/year	−0.713 *	−0.739 **	−0.732 *	−0.706 *	−0.640 *	−0.520	−0.295	−0.195	0.147	0.362
Replant 145,000 hectares/year	−0.712 *	−0.739 **	−0.731 *	−0.702 *	−0.627 *	−0.492	−0.237	−0.116	0.282	0.418
Replant 150,000 hectares/year	−0.712 *	−0.740 **	−0.730 *	−0.697 *	−0.614 *	−0.462	−0.176	−0.038	0.345	0.518

* Correlation is significant at α = 0.05. ** Correlation is significant at α = 0.01.
